# Supplementary material for: Functional characterization of C21ORF2 association with the NEK1 kinase mutated in human in diseases
Source: Life Sci Alliance. 2023 May 15;6(7):e202201740. doi: 10.26508/lsa.202201740 (PMC10185812; doi:10.26508/lsa.202201740)
Supplement: Supplementary file 1 [file LSA-2022-01740_SdataF1_F3_F4_FS2.2_FS3_FS4_FS5_FS6_FS7.pdf]

1A

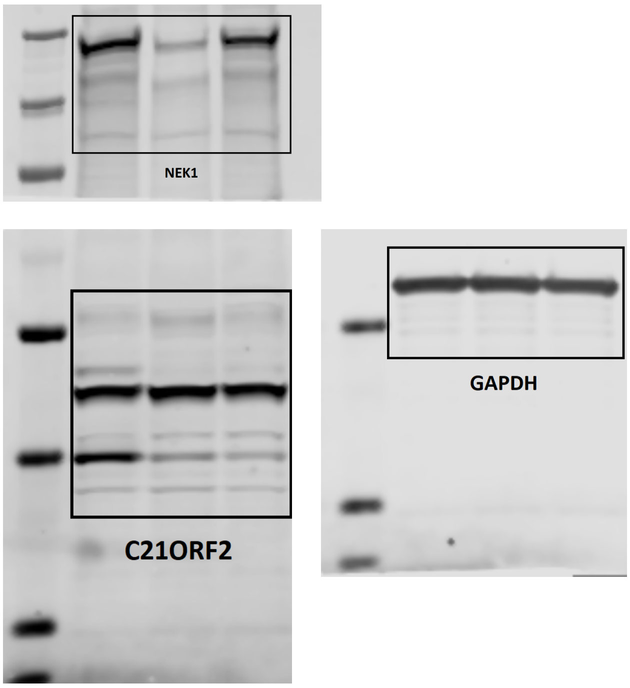

1B

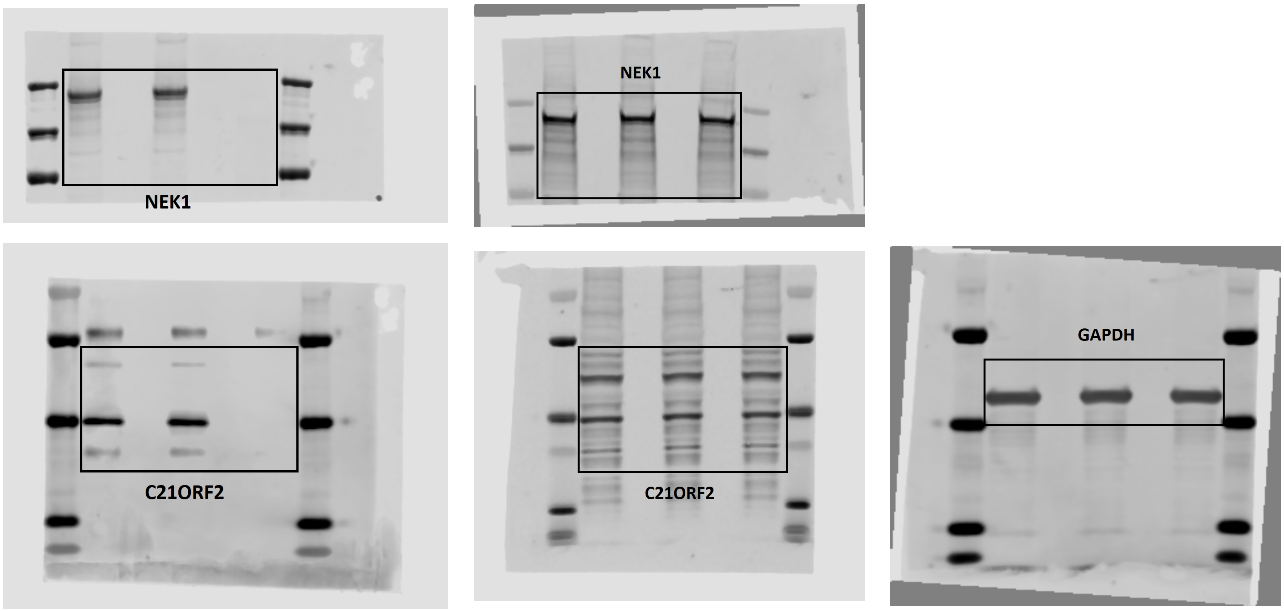

1C

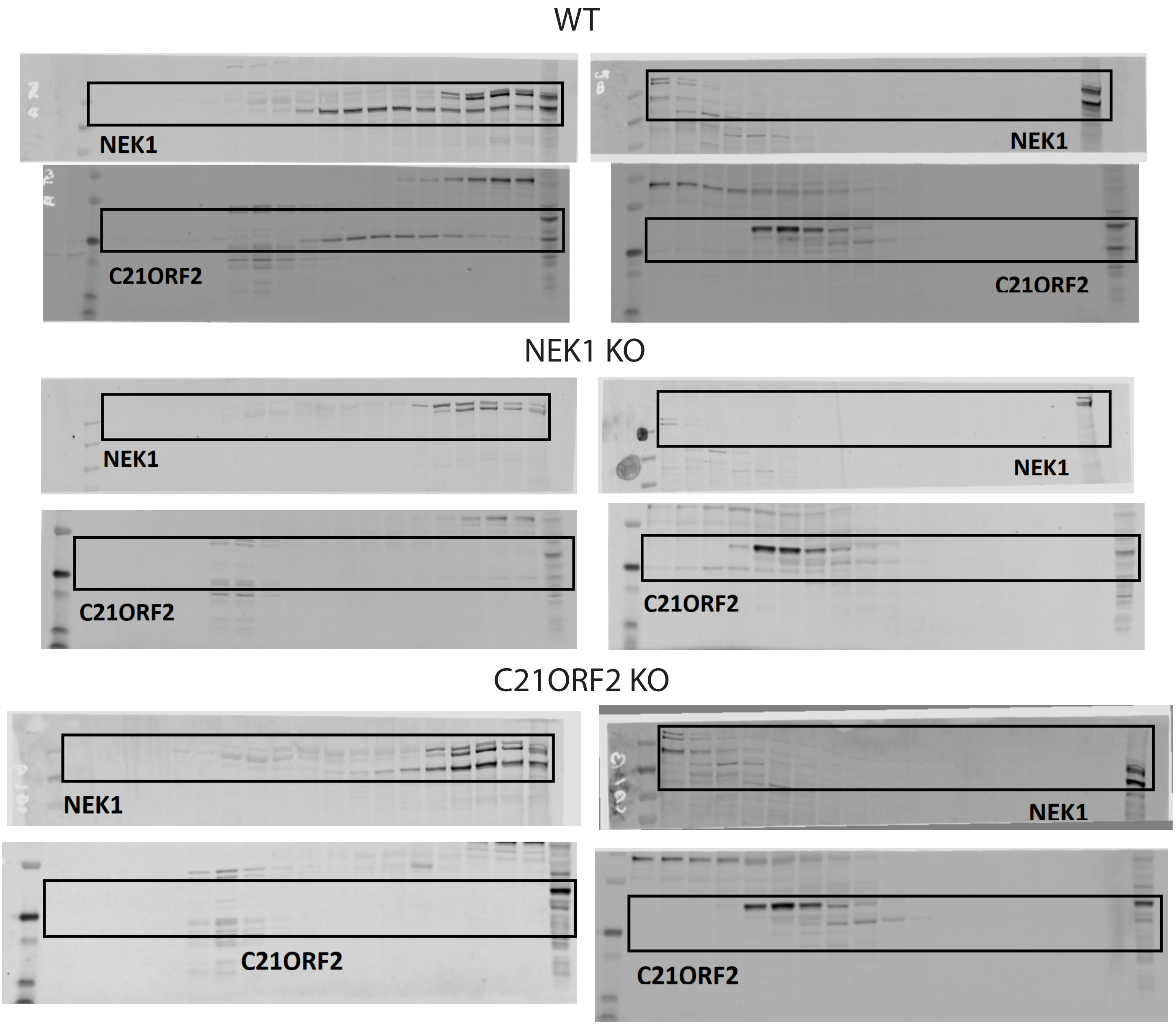
WT
NEK1
NEK1
C21ORF2
C21ORF2
NEK1 KO
NEK1
NEK1
C21ORF2
C21ORF2
C21ORF2 KO
NEK1
NEK1
C21ORF2
C21ORF2

1D

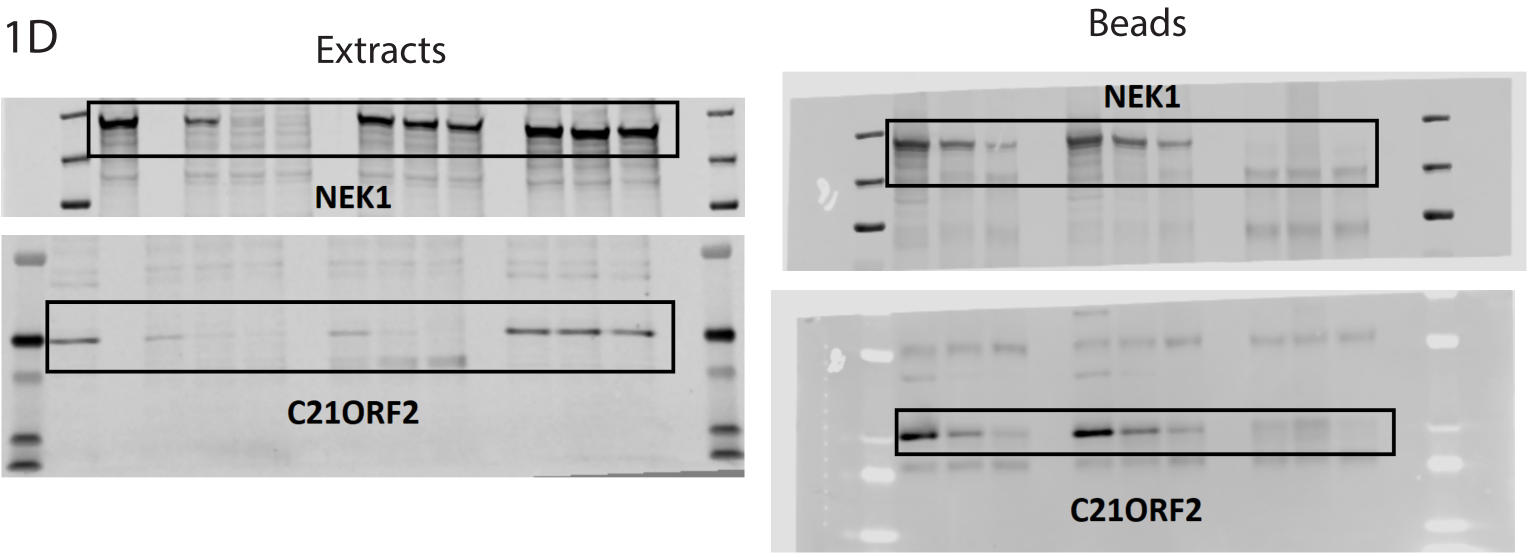
Extracts
NEK1
C21ORF2
Beads
NEK1
C21ORF2

3A

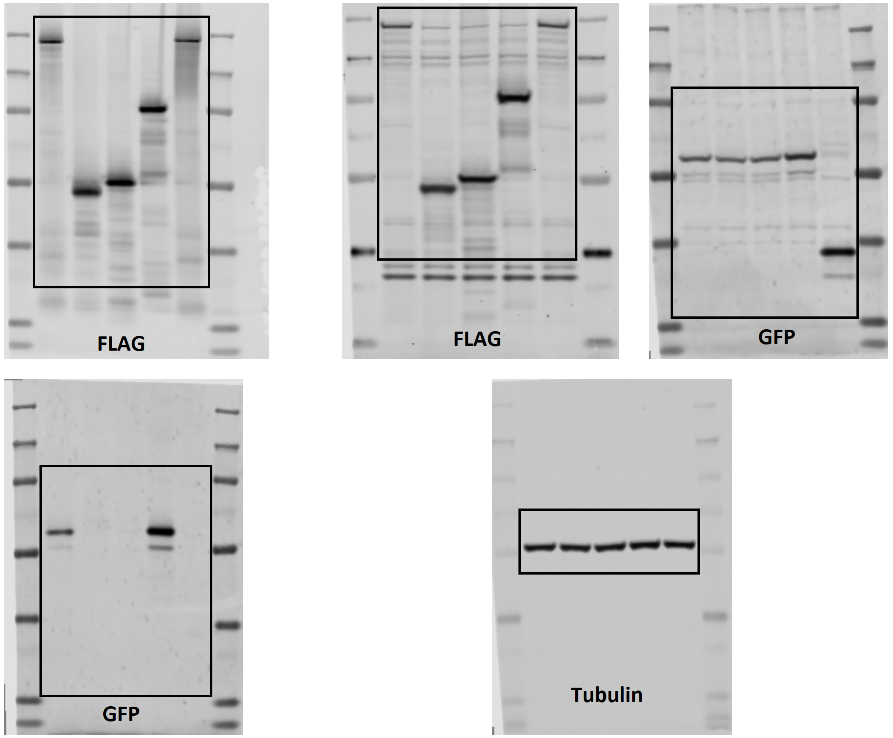
FLAG
FLAG
GFP
GFP
Tubulin

3B

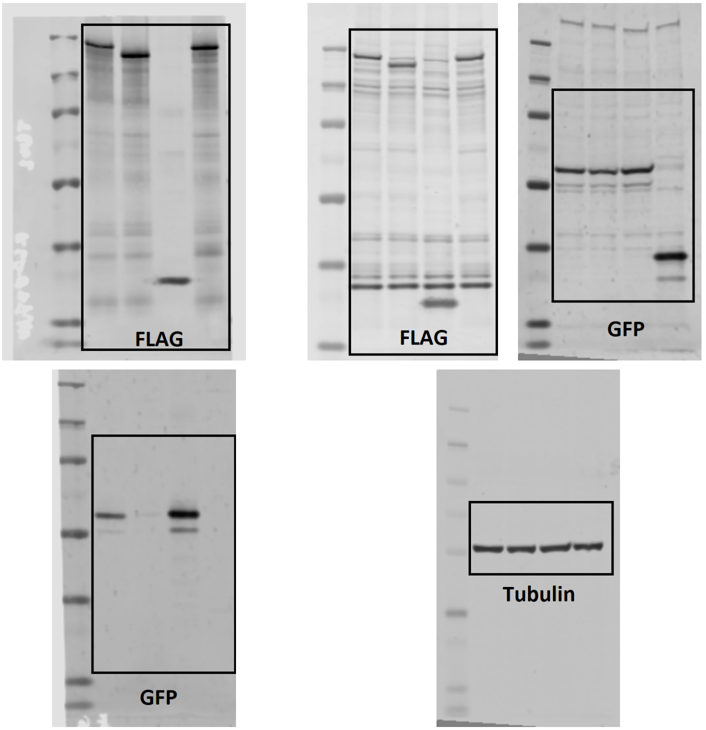
FLAG
FLAG
GFP
GFP
Tubulin

3C

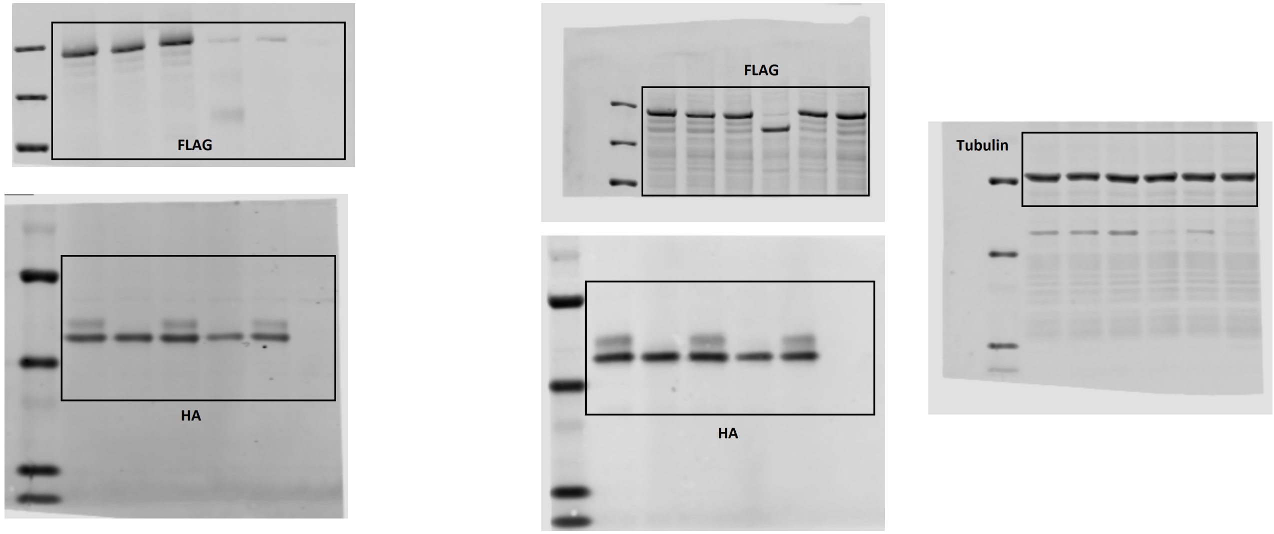
FLAG
HA
FLAG
HA
Tubulin

4D

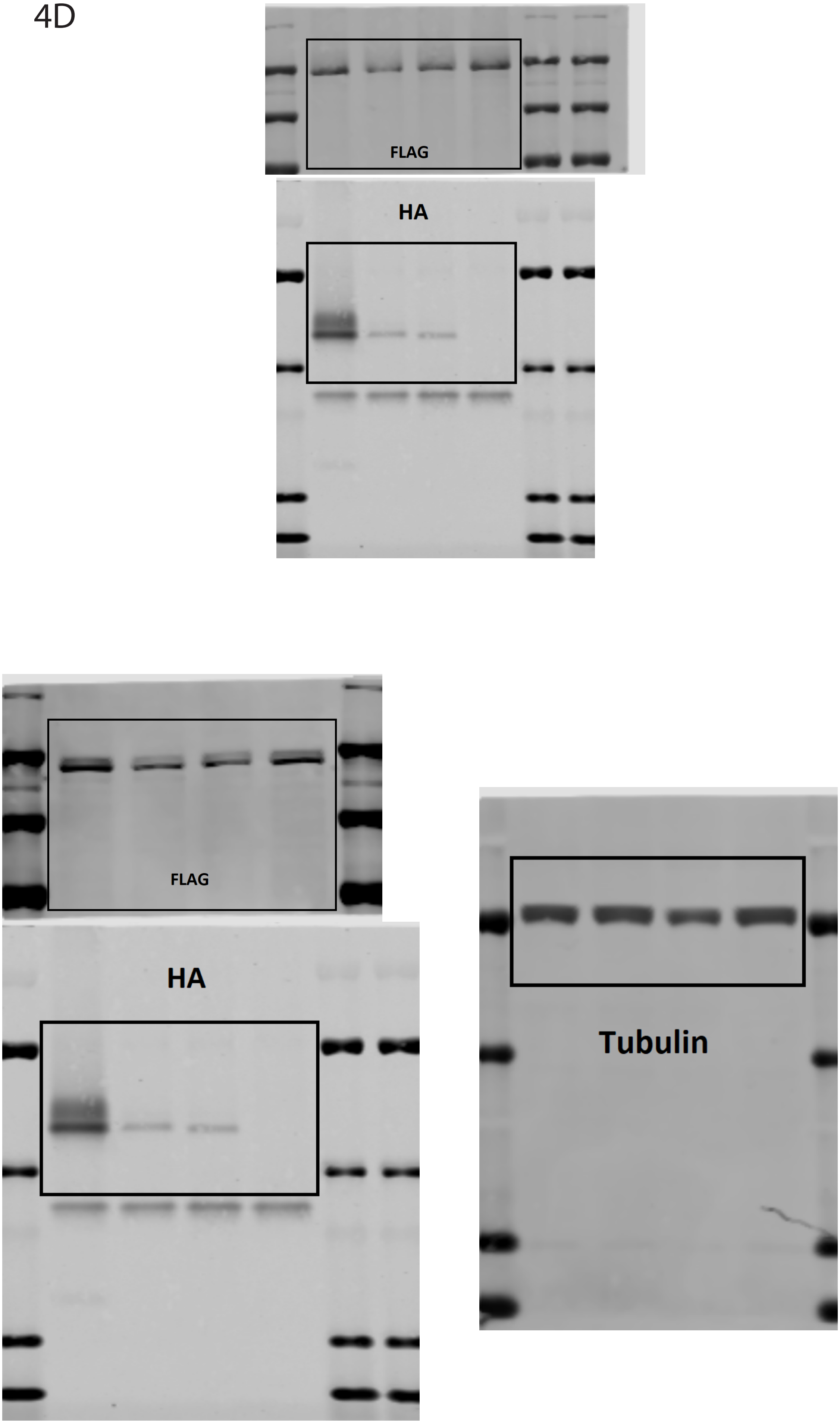

S2A and S2B

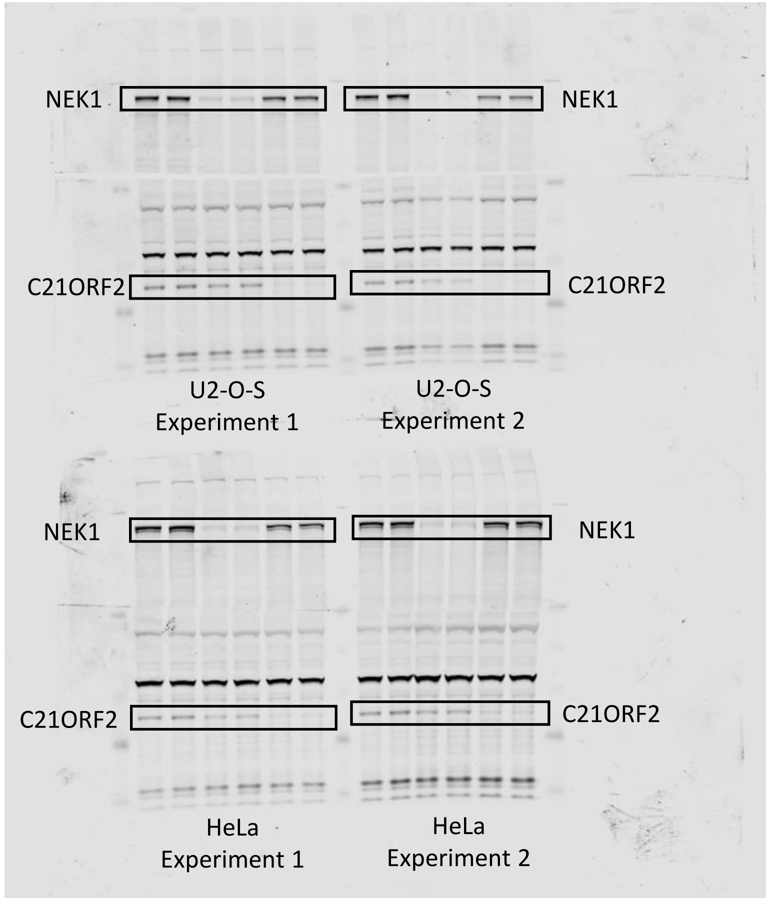
NEK1
NEK1
C21ORF2
C21ORF2
U2-O-S
Experiment 1
U2-O-S
Experiment 2
NEK1
NEK1
C21ORF2
C21ORF2
HeLa
Experiment 1
HeLa
Experiment 2

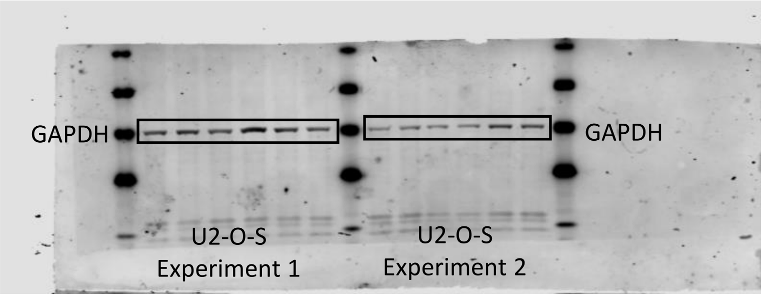
GAPDH
GAPDH
U2-O-S
Experiment 1
U2-O-S
Experiment 2

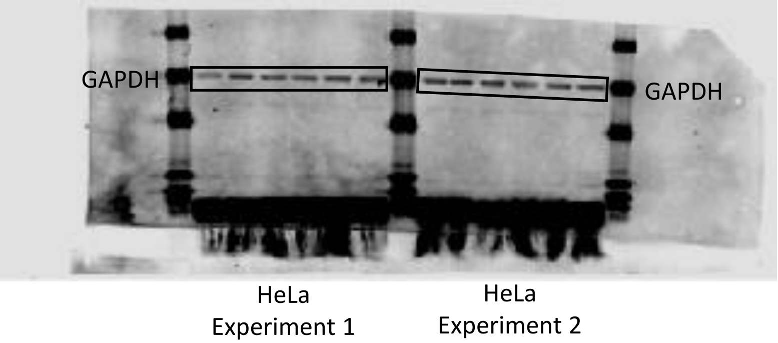
GAPDH
GAPDH
HeLa
Experiment 1
HeLa
Experiment 2

S2C MG-132

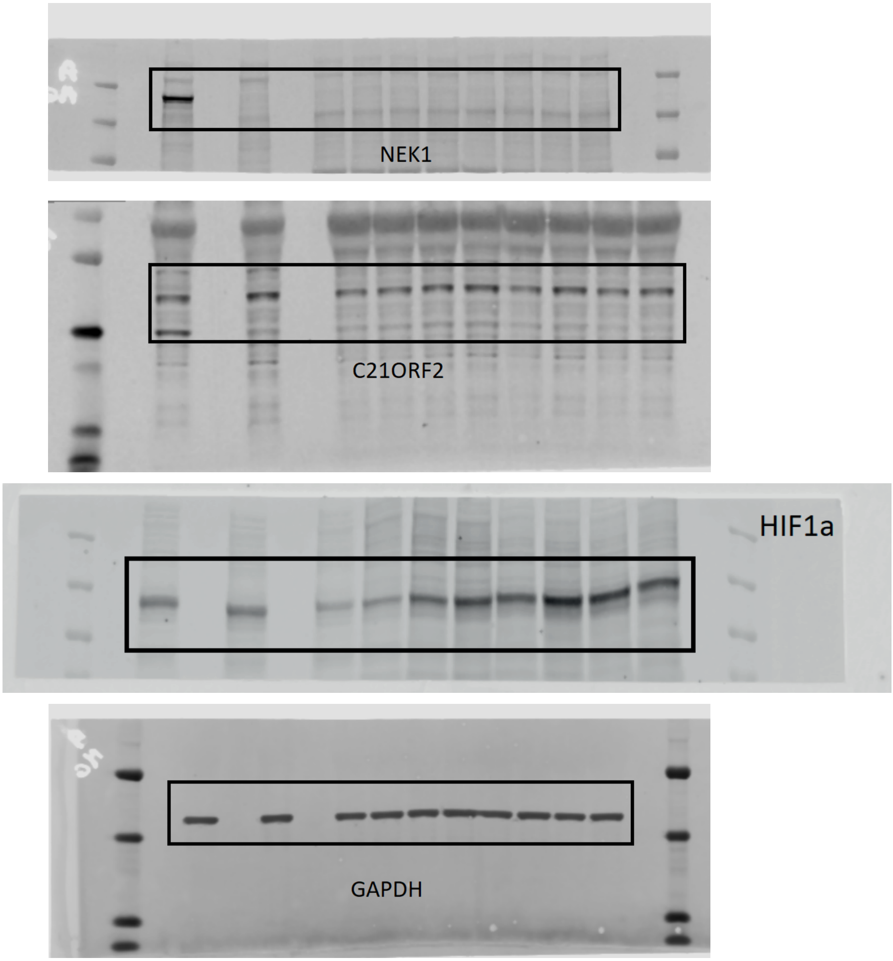

S2D MLN-4792

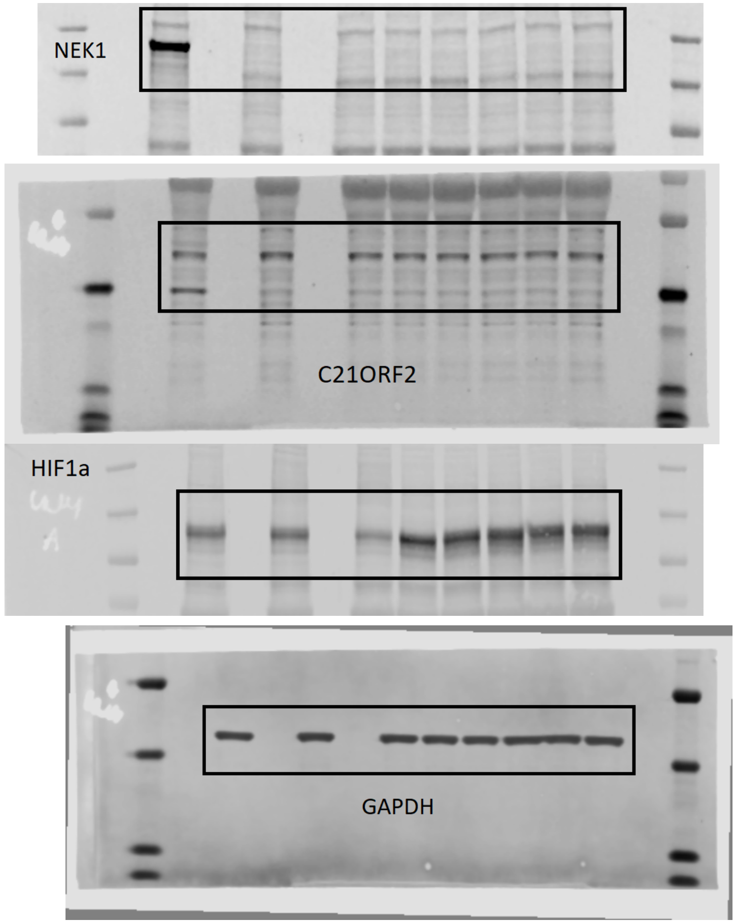

S2E BafA1

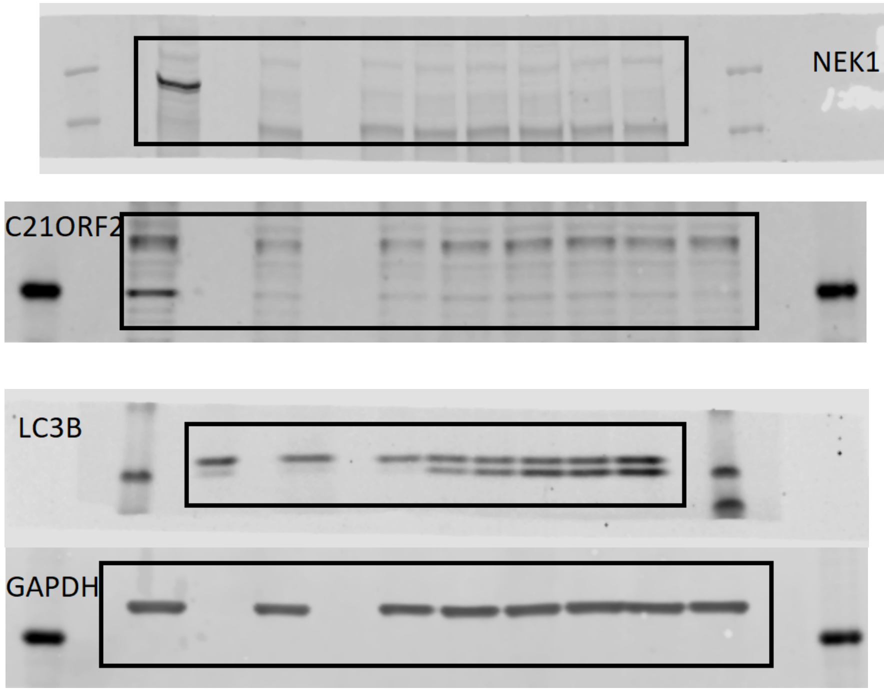

S2F MRT68921

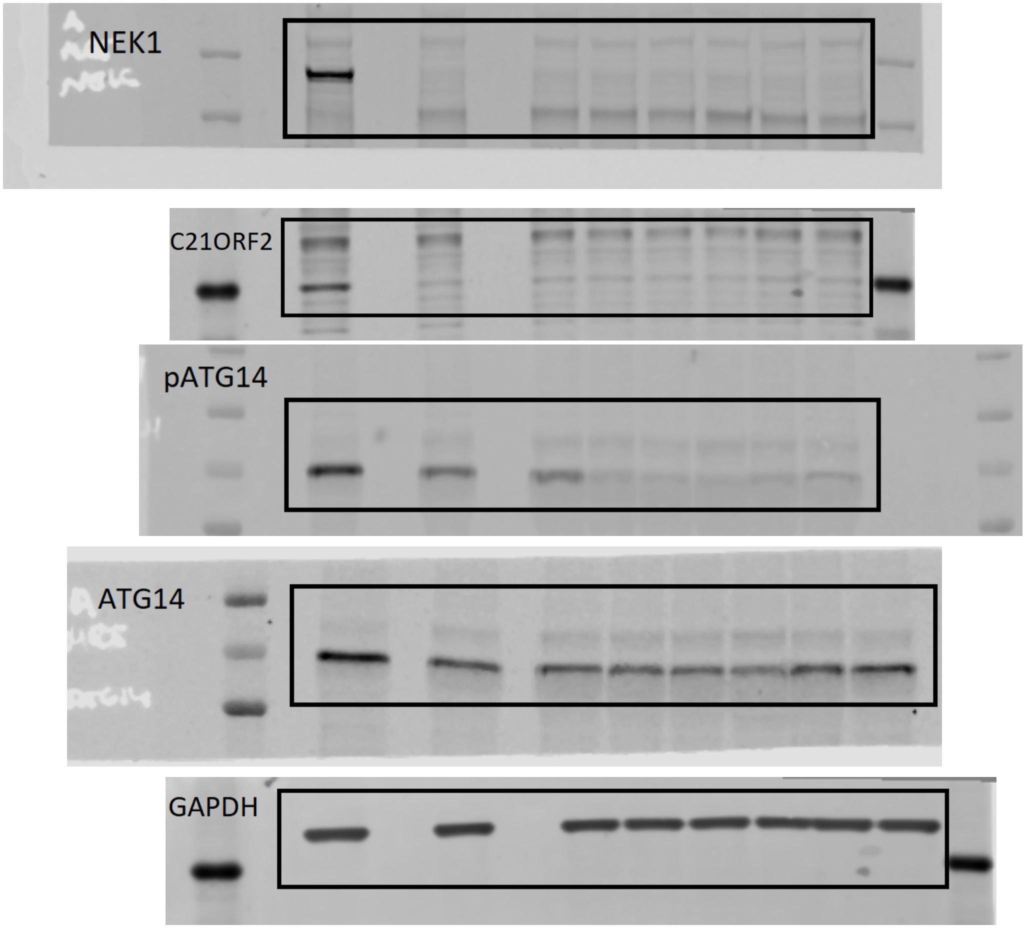

S3A

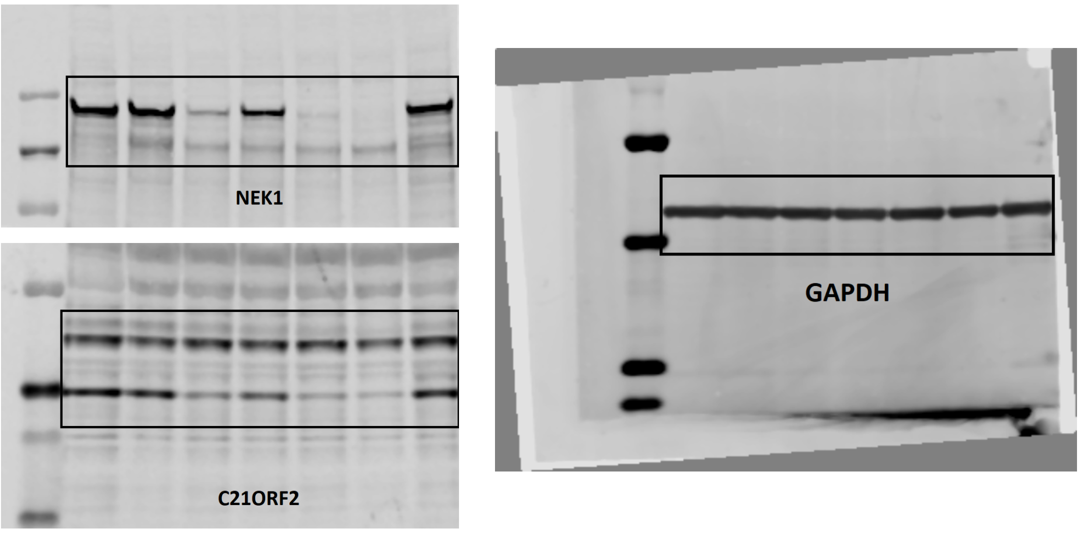

S3B

NEK1 IP

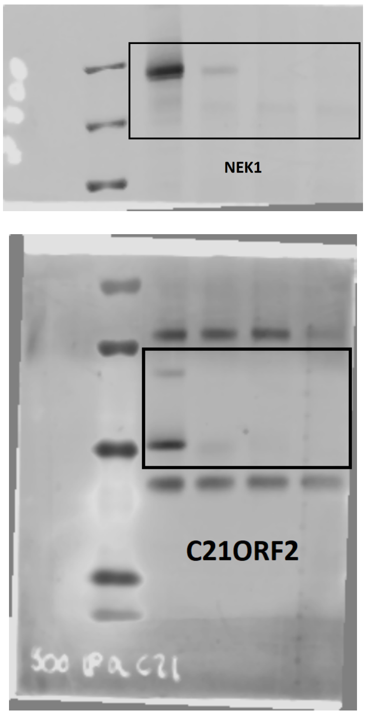

C21ORF2 IP

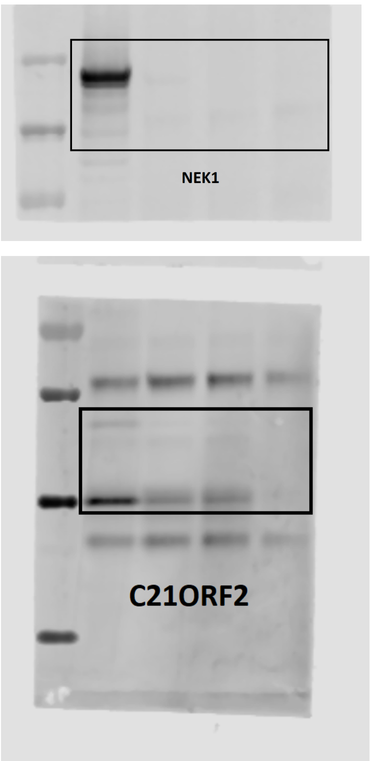

Input

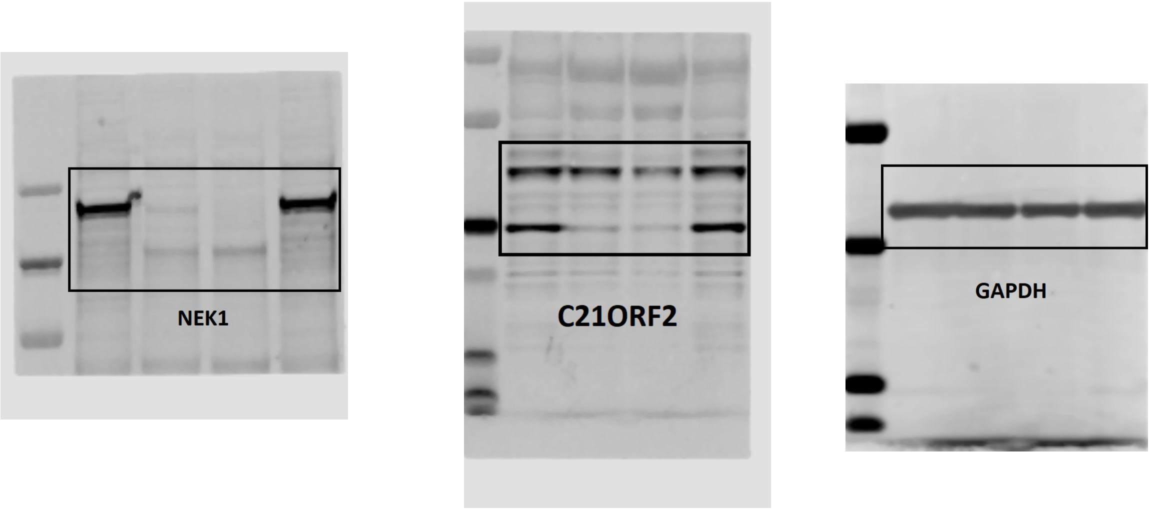

S4A

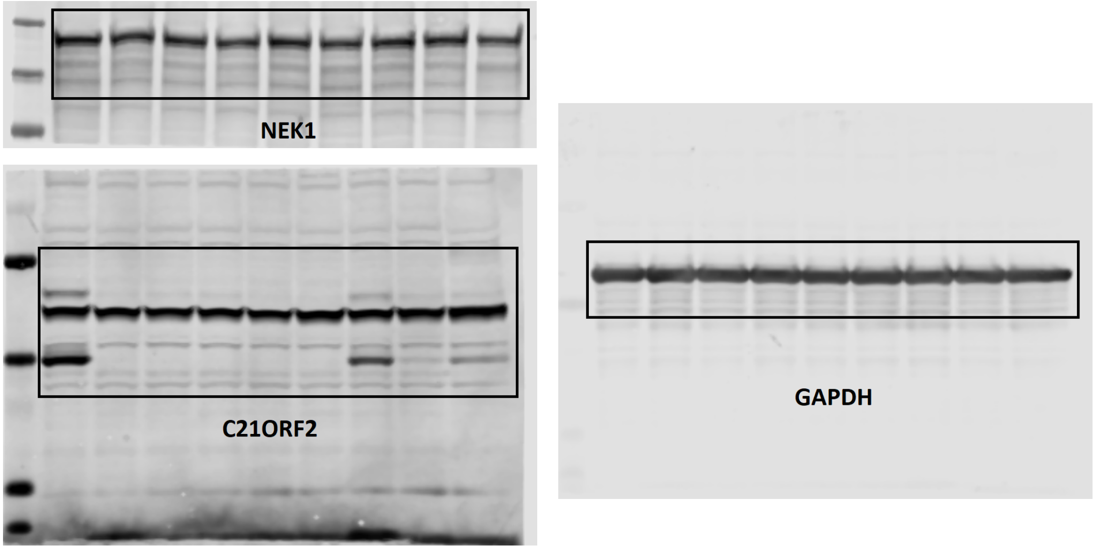
NEK1
C21ORF2
GAPDH

S4B

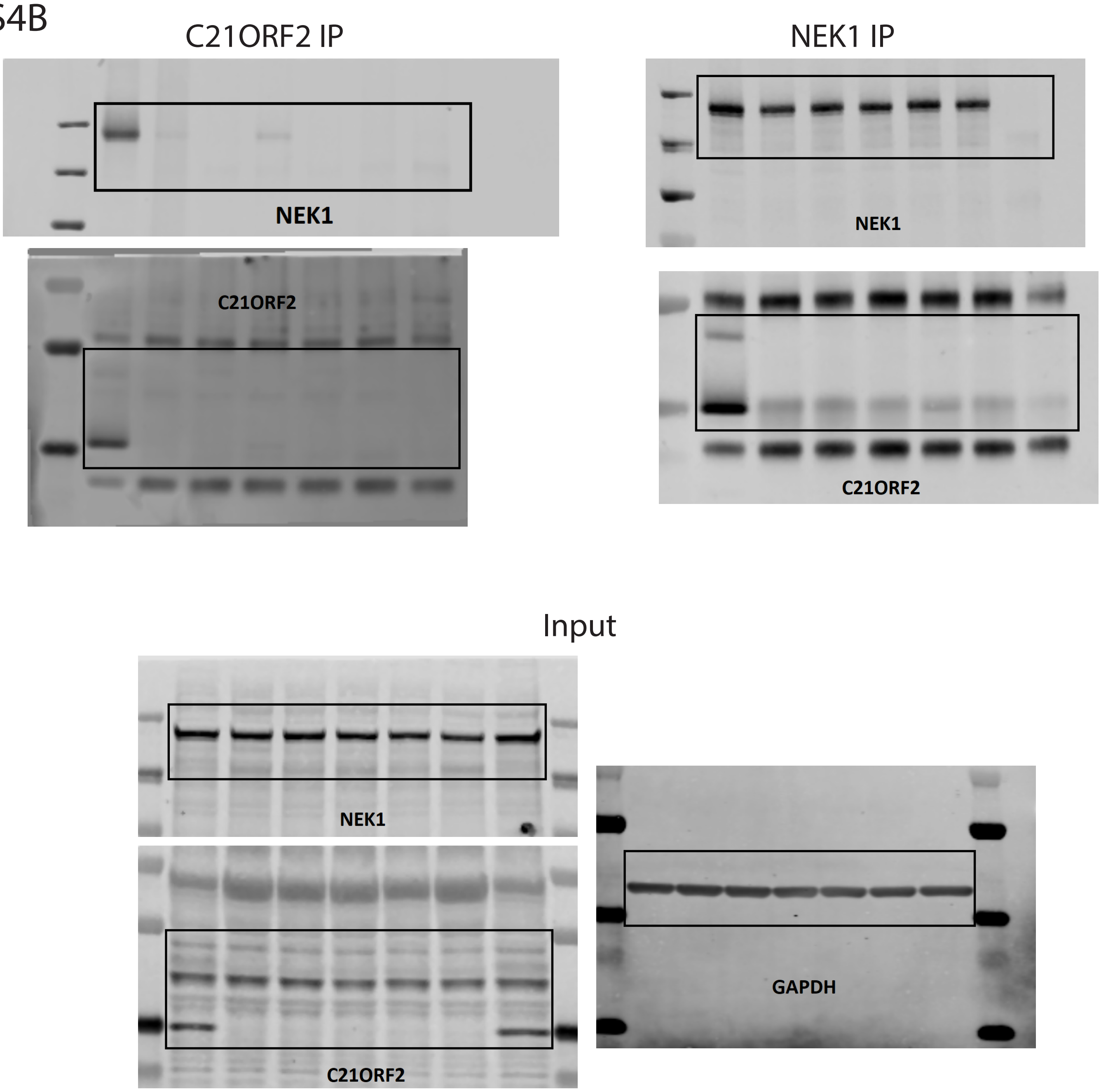
C21ORF2 IP
NEK1 IP
NEK1
C21ORF2
NEK1
C21ORF2
Input
NEK1
C21ORF2
GAPDH

S5

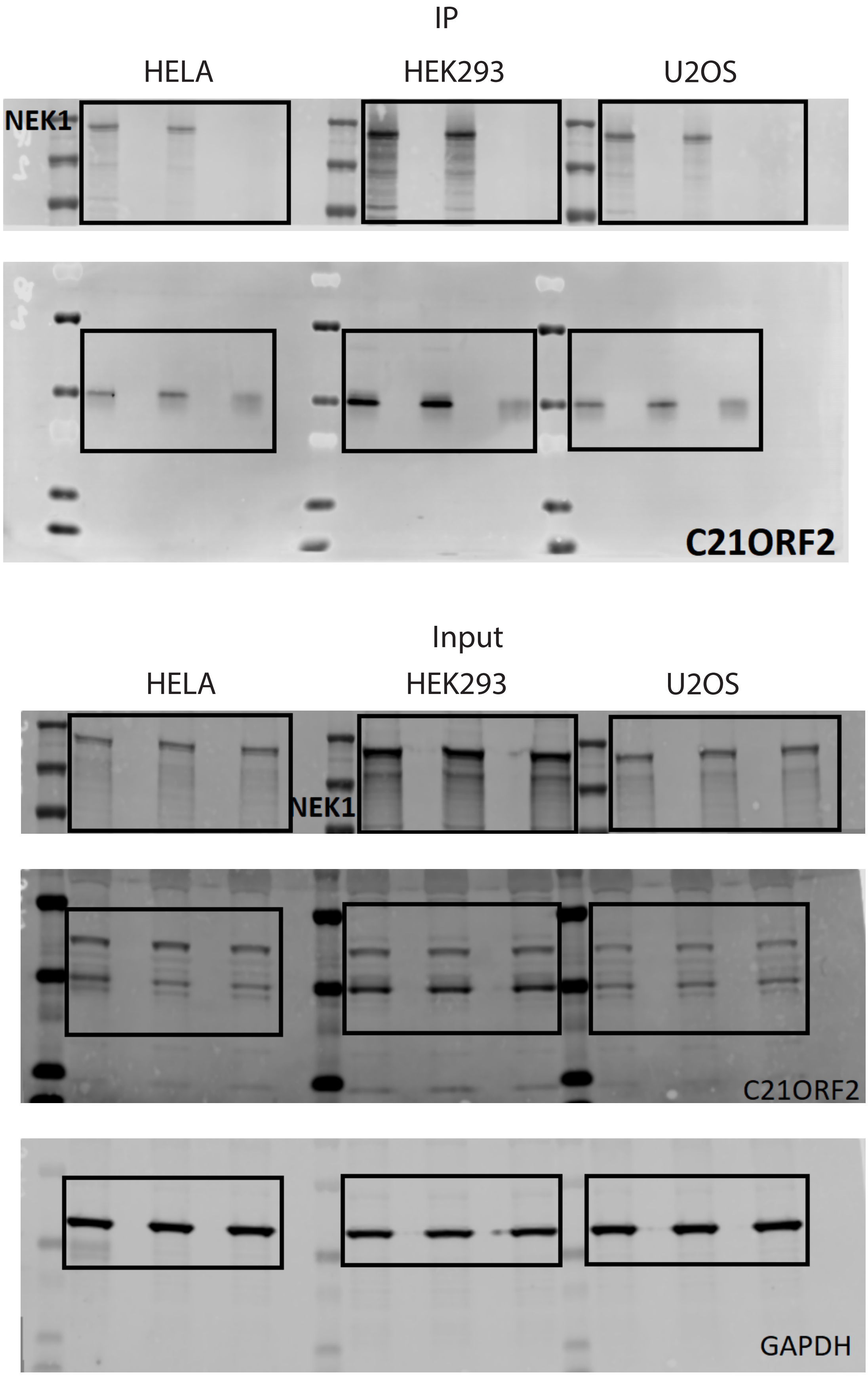
IP
HELA
HEK293
U2OS
NEK1
C21ORF2
Input
HELA
HEK293
U2OS
NEK1
C21ORF2
GAPDH

S6C

FLAG IP

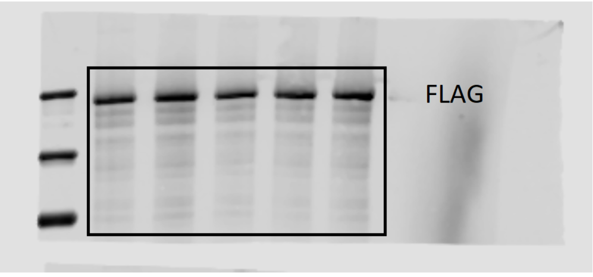

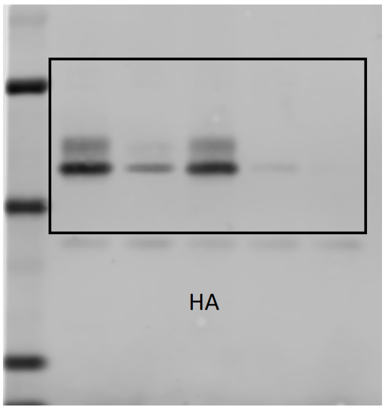

HA IP

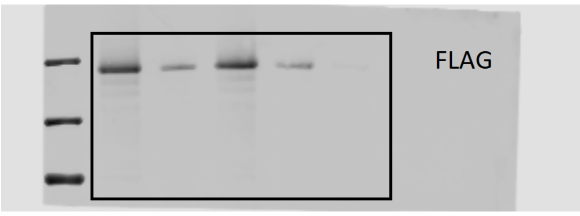

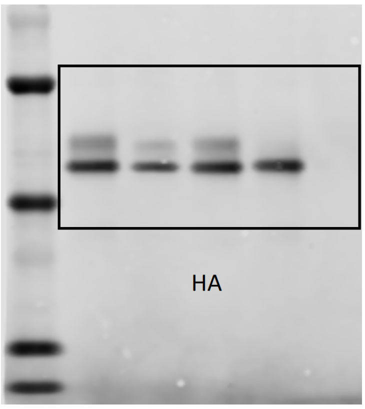

Input

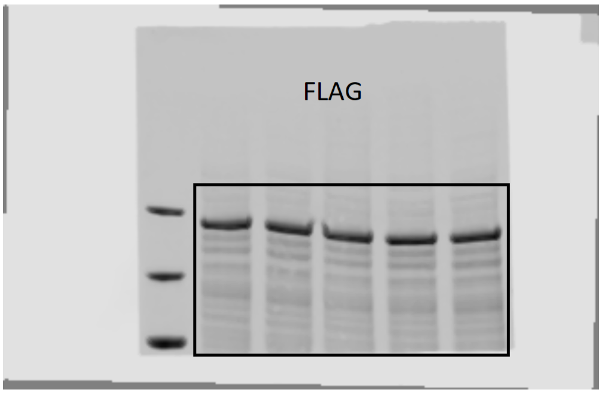

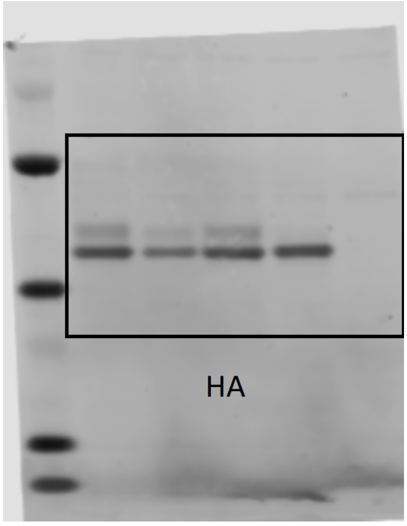

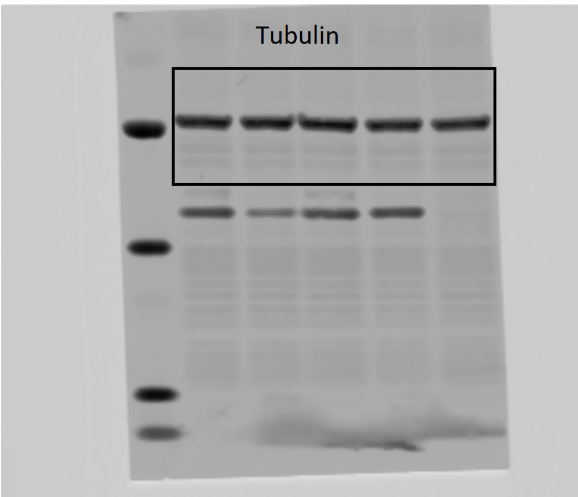

S7B

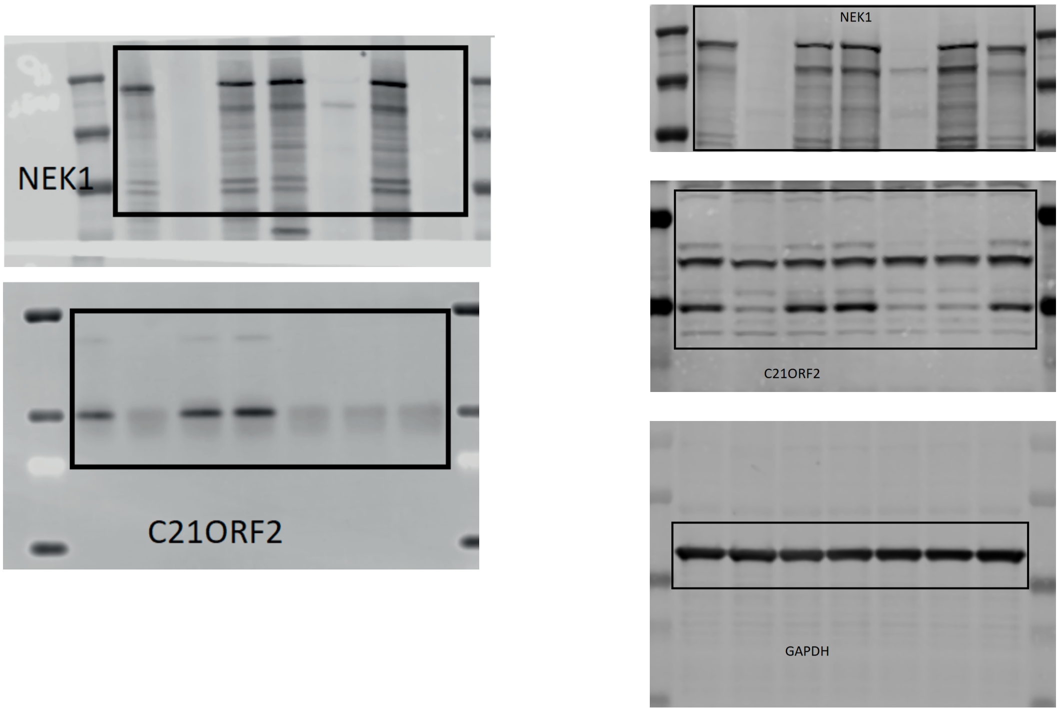
NEK1
C21ORF2
NEK1
C21ORF2
GAPDH

S7C

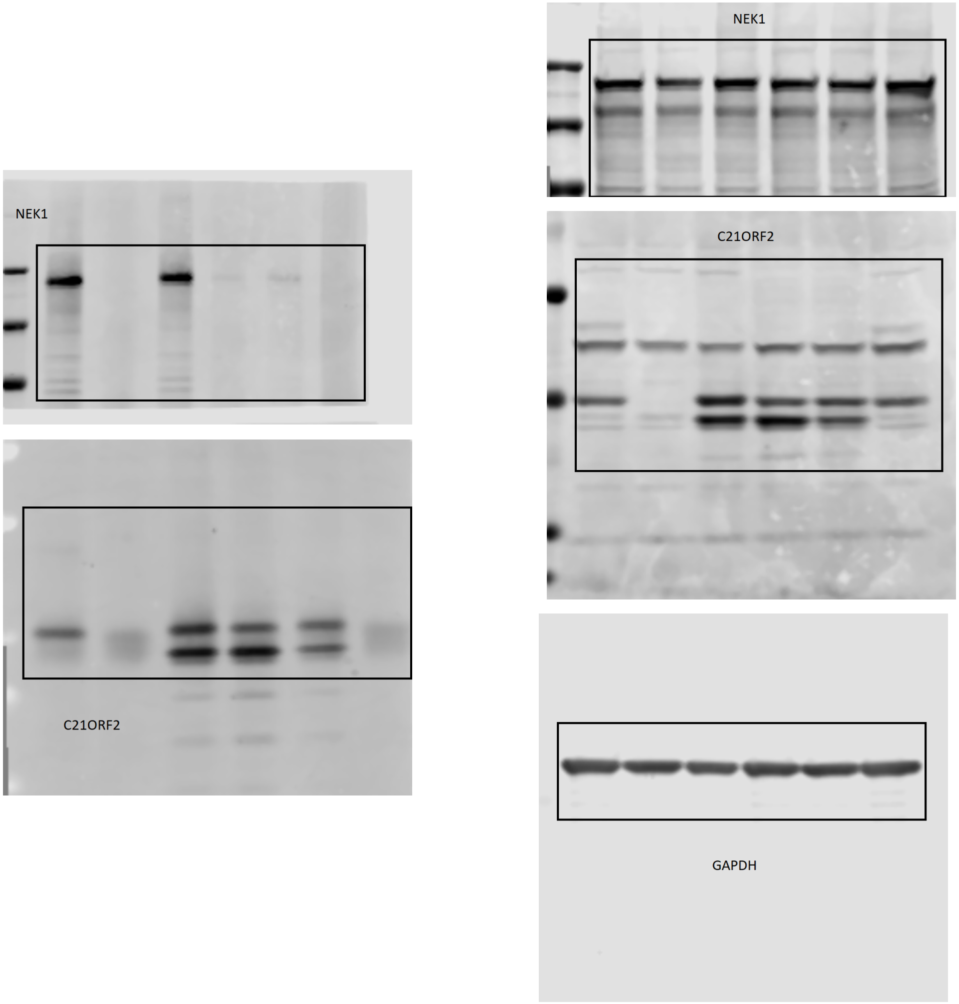
NEK1
C21ORF2
NEK1
C21ORF2
GAPDH
